# Ligand-directed covalent labelling of adenosine receptors

**DOI:** 10.1007/s11302-025-10073-y

**Published:** 2025-02-24

**Authors:** Chloe Keduan Li, Karen Joan Gregory, Manuela Jörg

**Affiliations:** 1 Medicinal Chemistry Theme, Monash Institute of Pharmaceutical Sciences, Monash University, Parkville, Australia; 2 Drug Discovery Biology, Monash Institute of Pharmaceutical Sciences, Parkville, VIC 3052 Australia; 3 ARC Centre for Cryo-Electron Microscopy of Membrane Proteins, Monash University, Parkville, VIC 3052 Australia; 4 Centre for Cancer, Chemistry, School of Natural and Environmental Sciences, Newcastle University, Newcastle Upon Tyne, UK

**Keywords:** Adenosine receptors, Chemical probes, Click reactions, Fluorescence, Ligand-directed covalent labelling

## Literature summary

**Fig. 1:**
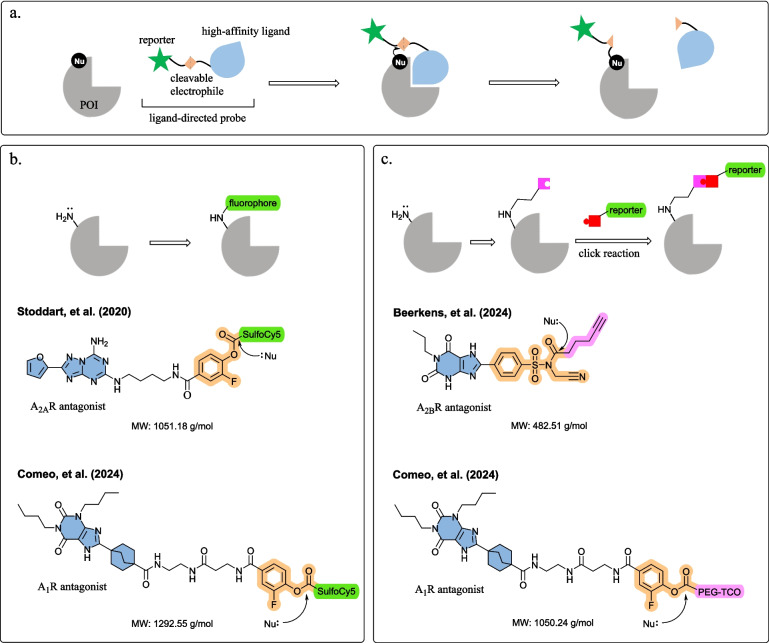
**a** Covalent labelling of POIs with ligand-directed probes. The process starts with the protein–ligand interaction, resulting in covalent cargo transfer from the probe to the target protein through a proximity-driven nucleophilic substitution reaction, followed by ligand dissociation from the binding pocket. Different types of ligand-directed probes targeting ARs were reported: **b** fluorophore conjugated ligand-directed probes and **c** click-handle conjugated ligand-directed probes. Colour labels: POI-ligand (blue), electrophilic warhead (orange), reporter tag (green), click handle (pink) and click partner (red)

Ligand-directed covalent labelling is an emerging method to label proteins of interest (POI). This method encompasses a POI ligand and a reporter moiety, connected via a reactive, cleavable electrophilic group (Fig. 1a) [1]. Upon binding, the electrophile reacts with a nucleophilic amino acid residue in close proximity, which irreversibly labels the POI with the reporter and releases the ligand [1, 2]. Unlike commonly used chemical probes, which occupy the protein binding site, the binding pocket remains accessible after ligand-directed covalent labelling, allowing further interrogation of the POI without disturbing or altering its native function [3]. Ligand-directed covalent labelling was first attempted to visualise enzymes, such as human carbonic anhydrase II (hCAII) and receptor protein FK506-binding protein 12 (FKBP12) [4]. Later, this method was applied to endogenous G protein-coupled receptors (GPCRs) in living cells to study localization, life cycle and ligand binding kinetics [5]. In this article, we discuss recent progress in ligand-directed adenosine receptor (AR) labelling that holds promise in studying adenosine signalling pathways under more physiologically relevant conditions. Locating endogenous ARs in living cells potentially aids in establishing new therapeutics targeting ARs for various diseases, including cardiovascular diseases, neurodegenerative disorders and cancers [6, 7]. The ligand-directed probes reviewed in this article (Fig. 1b and c) have used different strategies for AR fluorescent labelling: (1) ligand-directed probes conjugated with a fluorophore to irreversibly label ARs upon binding and (2) ligand-directed probes introducing a click handle for secondary incorporation of a fluorescent label. In 2020, Stoddart et al. [8] published the first ligand-directed probe to covalently and selectively label adenosine A_2A_ receptors (A_2A_Rs) with a sulfo-Cyanine5 (sulfo-Cy5) fluorophore. More recently, Comeo et al. [9] and Beerkens et al. [10] pioneered ligand-directed labelling of adenosine A_1_ receptors (A_1_Rs) and A_2B_ receptors (A_2B_Rs) respectively, functionalising the receptors with a bioorthogonal click chemistry handle, opening up new opportunities to quantitatively evaluate endogenous ARs in cell biology.

## Commentary

Adenosine signalling pathways have a significant role in many physiological processes involving immune response, regulation of vascular function and energy metabolism [6]. There are four distinct subtypes of adenosine G protein-coupled receptors (A_1_R, A_2A_R, A_2B_R and A_3_R) and all can be activated by the endogenous ligand adenosine [11]. A_2A_Rs primarily couple to the G_s_ protein that activates adenylyl cyclase, therefore increasing cyclic adenosine monophosphate (cAMP) levels [7, 12]. The A_2B_R subtype is positively coupled to adenylyl cyclase and phospholipase C through G_s_ and G_q_ protein coupling, respectively [7, 12]. In contrast, A_1_R couples to G_i/o_ proteins to inhibit adenylyl cyclase and A_3_R couples to both G_i_ and G_q_ proteins [7, 12]. Over the past decades, AR biology exploration has been supported by a diverse range of chemical probes, including radioligands, fluorescent and covalent ligands [12, 13]. Technological advances in fluorescence microscopy have enabled high-resolution fluorescent cell imaging, making fluorophores increasingly commonly used reporters in chemical probes to study biological processes [14]. However, there are still many challenges to studying adenosine signalling pathways. Molecular biology methods, such as fluorescent protein fusions or monoclonal antibodies, were extensively practiced in studying GPCRs, but have limitations, such as photobleaching and cell toxicity [15]. In addition, the wide AR distribution in the human body poses challenges in determining tissue-specific and systemic effects of AR ligands [16]. Therefore, we need more innovative chemical probes to reveal the mechanisms, life cycle and interaction of ARs in different cells, tissues and organs.

Stoddart et al. [8] and Comeo et al*.* [9] developed fluorophore-conjugated ligand-directed AR probes, which selectively and covalently labelled the AR subtype without affecting the binding site (Fig. 1b). Both fluorophore-conjugated probes comprising a high-affinity ligand (A_2A_R antagonist ZM241385 and A_1_R antagonist 8-bicyclo[2.2.2]octyl xanthine-based amino-functionalized congeners) and a sulfo-Cy5 fluorophore, connected via a small fluorine-substituted phenyl ester reactive linker [8, 9]. Upon binding, the phenyl ester reacts with a lysine residue near the binding site (K153 of A_2A_R or K168 of A_1_R) and the sulfoCy5 fluorophore was transferred to the receptor [8, 9]. To explore probe anchoring, both Stoddart et al. [8] and Comeo et al*.* [9] conducted molecular docking to reveal close proximity between the electrophile phenyl ester and the lysine residues K153 and K168 on the receptor surface (4 − 7 Å and 4 − 6 Å, respectively). Stoddart et al. [8] showed the ligand-directed A_2A_R probes successfully labelled endogenously expressed A_2A_R in live cell confocal imaging. Comeo et al*.* [9] observed agonist-induced A_1_R internalisation in response to agonist stimulation. Visualising receptor internalisation with ligand-directed fluorescent probes provides scope to assay novel AR ligands for effects on receptor trafficking. Moreover, the ligand-directed labelling approach may provide opportunities to visualise localisation and trafficking of adenosine heteroreceptor complexes, for instance A_2A_R and dopamine D_2_ receptors heteromers, to support novel drug discovery for Parkinson’s disease and schizophrenia [17–19].

Comeo et al. [9] and Beerkens et al. [10] developed ligand-directed probes with a reactive click handle, labelling the receptors in two steps (Fig. 1c). First, the click handle was covalently tagged to the receptor with ligand-directed chemistry, then a labelling moiety such as fluorophore was installed via click chemistry [9, 10]. Bioorthogonal chemistry represents a series of reactions that can occur within living systems without disrupting native biochemical processes, often utilizing click chemistry due to its rapid, efficient and high-yielding nature [20]. The click chemistry approaches reduce ligand-directed probe size, which might improve probe accessibility and specificity for the target protein as well as physicochemical properties such as solubility and permeability. Comeo and colleagues [9] described a polyethylene glycol trans-cyclooctene (PEG-TCO) click handle equipped probe, where a copper-free inverse electron demand Diels–Alder (IEDDA) click reaction was performed to attach a tetrazine-conjugated turn-on fluorophore on A_1_Rs. The fluorescence was visualised through in-gel fluorescence in recombinant cells stably expressing tagged A_1_Rs (human and rat) [9]. Beerkens et al. [10] engineered a clickable ligand-directed A_2B_R probe with a lysine-reactive *N*-acyl-*N*-alkyl sulfonamide (NASA) electrophilic warhead. After covalently transferring the alkyne click handle onto K267 or K269 of A_2B_R, copper-catalysed azide-alkyne cycloaddition (CuAAC) chemistry installed azide-conjugated reporter tags (N_3_-Cy5 and N_3_-biotin) [10, 20]. The probe bound to and labelled A_2B_R as observed in sodium dodecyl sulfate–polyacrylamide gel electrophoresis (SDS-PAGE), flow cytometric and mass spectrometry [10]. Turn-on fluorophores usually yield high signal-to-noise ratios overcoming the limitation of high fluorescent background noise when using conventional fluorescent probes in living cells [21]. Additionally, this method offers opportunities to label the protein with different reporter tags as best fit for purpose, including fluorophores with different spectral properties or biotin for pull-down or purification [10]. One should keep in mind that copper might pose cytotoxicity problems, limiting its uses in live cells; therefore, copper-free click chemistry reactions, such as Staudinger ligation reaction, strain-promoted alkyne-azide cycloaddition (SPAAC), strain-promoted alkyne-nitrone cycloaddition (SPANC) or IEDDA reaction, are generally more suitable for in vitro and in vivo applications [20, 22].

An essential element in ligand-directed chemistry is selecting electrophilic groups that bear a balance between labelling efficiency and selectivity: electrophilic groups should be reactive to form covalent bonds with often poorly nucleophilic amino acid side chains, but also target- and site-specific for selective labelling [9]. Stoddart et al. [8] and Comeo et al. [9] employed a fluorine-substituted phenyl ester group targeting lysine residues in the binding pocket. To increase the electrophilic groups’ reactivity, an electron-withdrawing fluorine atom was installed adjacent to the phenyl ester group. The small-sized fluorine-substituted phenyl ester fits in restricted AR binding pockets, and is optimal in reactivity to form a covalent bond [23]. Beerkens and colleagues’ successfully developed NASA sulfonamide probes from existing fluorosulfonyl-substituted covalent ligands to label A_2B_Rs. NASA electrophiles have the fastest reaction rate for modifying a lysine residue compared to other commonly used ligand-directed electrophiles [24]. Incorporating a cyano moiety to the NASA group improved reactive kinetics between the ligand and the A_2B_R. However, the cyano-substituted NASA system has high intrinsic reactivity, which may be responsible for the observed non-specific labelling [8]. Therefore, the authors suggested further optimisation, for instance replacing the cyano moiety with 1,3-difluorobenzene or 3-fluoropyridine, as evident in the latest study by Hamachi’s group [25]. The fluorosulfonyl warhead is commonly incorporated into covalent drug discovery and chemical probes, owing to the excellent reactive kinetics, stability in water and selectivity towards nucleophilic amino acid residues, including cysteines, tyrosines and lysines [26]. There are multiple established fluorosulfonyl-containing AR probes, such as A_1_R antagonist DU172 [27], A_2A_R antagonist LUF7445 [28], A_3_R antagonist LUF7602 [29] and irreversible ectonucleotidase inhibitor 5′-p-fluorosulfonyl benzoyl adenosine (5′FSBA) [30]. Therefore, this approach has great potential to evolve existing fluorosulfonyl-containing probes to enable ligand-directed labelling across different purinergic receptors and readily translatable to other protein classes for which fluorosulfonyl covalent ligands exist.

In conclusion, the three studies reviewed herein have validated ligand-directed labelling approaches for studying ARs. The ligand-directed covalent probes introduced in these studies offer significant advantages, such as (1) labelling ARs without requiring genetic modification and (2) allowing the ligand to dissociate post-labelling, preserving the native function of ARs. In particular, the development of NASA sulphonamide probes from established literature fluorosulfonyl-containing covalent probes provides a straightforward strategy for quickly developing novel ligand-directed purinergic probes. However, this technique still has limitations and has not yet found application in live tissues and broader organisms. To expand ligand-directed labelling in chemical biology and drug discovery, further optimisation is required in identifying subtype-selective ligands, ligand-directed chemistries and simple synthetic methods.

## Data Availability

No datasets were generated or analysed during the current study.
